# Association of Low Maternal Pregnancy-associated Plasma Protein A with Adverse Perinatal Outcome

**DOI:** 10.7759/cureus.4912

**Published:** 2019-06-17

**Authors:** Panagiotis Antsaklis, Zacharias Fasoulakis, Marianna Theodora, Michail Diakosavvas, Emmanuel N Kontomanolis

**Affiliations:** 1 Obstetrics and Gynecology, National and Kapodistrian University of Athens, Athens, GRC; 2 Obstetrics and Gynecology, Democritus University of Thrace, Alexandroupolis, GRC

**Keywords:** papp-a, adverse perinatal outcome, iugr, preeclampsia, stillbirth, down syndrome

## Abstract

The aim is to provide an overall view of the association of low pregnancy-associated plasma protein A (PAPP-A) levels with adverse perinatal outcomes. The available literature in PubMed/Medline regarding PAPP-A and adverse pregnancy outcomes was searched for related articles, including terms such as “PAPP-A,” “intrauterine growth restriction (IUGR),” “small for gestational age (SGA),” “stillbirth,” “adverse outcome,” and others. The fifth percentile is supported by many recent studies to be PAPP-A’s cutoff for adverse outcome detection and the increased risk seems to be extremely high below 0.2 PAPP-A MoM (multiple of the median). Apart from chromosomal abnormalities, preeclampsia, intrauterine fetal demise, and pregnancy loss have been associated with maternal serum PAPP-A. For results below the first centile, PAPP-A has a strong positive predictive value for SGA and IUGR. Except for its vital role on the cleavage of insulin-like growth factor binding proteins (IGFBP), PAPP-A has proven to be a reliable marker for prenatal screening. Even though PAPP-A as a single predictor proved to be valuable for the prediction of some adverse perinatal outcomes, in some cases, a combination of PAPP-A to other maternal serum markers led to an increase in detection rates. PAPP-A is a promising maternal serum marker for pregnancy outcome prediction with more studies needed in order for its potentials to be fully understood and exploited.

## Introduction and background

The prevention and prognosis of adverse pregnancy outcomes are of great significance and, from a medical point of view, particularly challenging for the scientific community, as well as for the family and society. In addition to parental information and psychopathology, socioeconomic factors represent the main cause for creating a reliable prenatal prognostic protocol. The need for early detection and prevention of adverse perinatal outcome is necessary nowadays.

Until today, nuchal translucency and second-trimester routine ultrasound still remain the best predictors for fetal aneuploidies [1]. However, combining fetal ultrasonography to pregnancy-associated plasma protein A (PAPP-A) leads to better diagnosis and prognosis of such anomalies, making them both compulsory for first-trimester prenatal screening in developed countries.

## Review

The role of PAPP-A

PAPP-A was detected 40 years ago in the plasma of pregnant women and was immediately suspected to have several biological roles due to similarities to other carrier and modulator proteins [2]. The role of PAPP-A in pregnancy was described soon thereafter, and since then, many studies have focused on the prediction of fetal anomalies with the help of this marker.

PAPP-A is one of the two pappalysins, a protease that belongs to the superfamily of metzincin (such as astacins, adamalysins, serralysins, the matrix metalloproteinases, and the pappalysins) of metalloproteinases and participates in the local release of insulin-like growth factors. PAPP-A consists of a heterotetrameric disulfide bound 2:2 complex composed of two 200 kDa PAPP-A subunits the 206-residue proform of eosinophil major basic protein (proMBP) subunits and has been assigned to human chromosome 9q 33.1. ProMBP functions as a proteinase inhibitor of PAPP-A. During pregnancy, these two glycosylated subunits are produced separately and then united secondarily. Although the PAPP-A mRNA can be found in many tissues, the placental production exceeds any other. The syncytiotrophoblast composes the PAPP-A subunit, while proMBP is synthesized in extravillous trophoblasts, and the two subunits are combined to create the final complex at the extracellular environment [3].

The role of insulin-like growth factor-I (IGF-I) and IGF-II is regulated by binding to one of six IGF binding proteins (IGFBP). PAPP-A is a highly selective enzyme and the responsible proteinase for cleavage of IGF binding proteins (specifically, IGFBP-4, IGFBP-5, and to some extent, IGFBP-2), acting as a regulator of IGF’s bioavailability. PAPP-A2, the second member of the pappalysin family, cleaves only IGFBPs 3 and 5. IGFBP is proteolyzed by PAPP-A, the bioavailability of IGF is enhanced, trophoblast invasion is mediated, and glucose and amino acids transport in the placenta is determined. PAPP-A is proven to be highly increased in blood serum of up to 100 and 10,000 times in the first and third trimesters of pregnancy, respectively, compared to the samples of nonpregnant women [4]. 

To date, there is no evidence of substrates, other than IGFBPs, that PAPP-A reacts with. However, except cleavaging insulin-like growth factors, several other studies about the biological role of PAPP-A have demonstrated a possible complementary role. Atherosclerosis was first to be correlated to increased PAPP-A in the 2001 study by Bayes-Genis et al. [5], and since then, several studies aimed at clarifying the connection between coronary syndrome and other atherosclerotic diseases to this metalloproteinase. Furthermore, increased levels of glomerular PAPP-A revealed an association with diabetic neuropathy, speculating a potential therapeutic role on inhibiting PAPP-A. Moreover, the involvement of PAPP-A in tumor development has been increasingly supported with an expression of PAPP-A in ovarian, breast, lung, and other cancer tissues constantly being reported [6].

Prediction of adverse pregnancy outcome

In early gestation, the placental function is believed to be mainly represented by first trimester analytes. By the 1990s, the introduction of improved biophysical monitoring techniques led many clinical studies to examine the relevance of PAPP-A to different pathological states during pregnancy and evaluating its reliability as a prognostic factor. Especially over the last two decades, the development of pioneer and affordable equipment led to wider access to available technological means. Research became more targeted and better recording led to more reliable results, and by measuring maternal serum PAPP-A levels and evaluating the results using the multiple of the median (MoM), each individual pregnant woman was able to be selected for a tighter monitoring until childbirth and to be informed on the possibilities of adverse pregnancy outcomes.

Early studies

Cornelia De Lange Syndrome and Threatened Abortion

In 1983, Wastergaard et al. published a study of three cases reported with the Cornelia De Lange syndrome in which PAPP-A was absent from the maternal serum and trophoblastic tissue, while in the same year, abnormally low levels of PAPP-A were connected to threatened abortion in 51 patients with live fetuses by the time of sampling [7]. This result was also supported by Masson et al. in a study of 60 women presenting with vaginal bleeding in the first trimester of pregnancy [7-8].

Anembryonic Pregnancies

Other studies reported that PAPP-A could be used as a predictor for anembryonic pregnancies. In 1984, Chapman et al. reported 22 cases with normal PAPP-A levels in the eighth week, while in 1986, out of 85 pregnancies, Yovich et al. presented 16 anembryonic pregnancies with low maternal serum PAPP-A levels at the seventh week of gestation. The precondition for both studies was the normal endocrinology and histology of the placenta [9-10].

Ectopic Pregnancy

Many studies recorded low levels or even absence of first trimester serum PAPP-A during ectopic pregnancies; however, most of them supported that a combination of serum biomarkers led to safer and more reliable results [11-14]. Since serum PAPP-A concentrations are normally very low before seven weeks of gestation, when used as a single predictor, it is poorly discriminative between ectopic pregnancy and other adverse outcomes.

Pregnancy Loss

A relationship between low levels of PAPP-A and pregnancy loss has been demonstrated in many studies [15-17]. Goetzl et al. presented a study of 7,932 pregnancies where low PAPP-A results were individually associated with increased early loss (even after logistic regression analysis for maternal age and race), while normal values presented a low risk of pregnancy loss [15]. Another study on developing patient-specific risk for fetal loss was presented in 2008 by Dugoff et al. [16]. Out of 36,014 women from the First- and Second-Trimester Evaluation of Risk (FaSTER) trial, 421 had an adverse outcome, dividing the results into two groups according to the week of fetal loss (< 24 or > 24 weeks). The results indicated a high detection rate with all the biomarkers examined, even though PAPP-A wasn’t the only significant marker for fetal loss. In addition, Scott et al. reported in a 2009 study that when PAPP-A level is low (and even with normal karyotype), there is an increased risk of adverse outcome and fetal loss (12% for < 0.2 PAPP-A MoM) [17]. In all studies, PAPP-A levels below the 5th centile were connected to an adverse pregnancy outcome and increased risk of fetal loss [15-17].

Intrauterine fetal demise

Intrauterine death and subsequent delivery beyond the 20th week of gestation occur in about one in 160 pregnancies in high-income countries with no improvement over the past decades. Even though major risk factors are already known for stillbirth (obesity, age, smoking, small for gestational age (SGA), diabetes, hypertension, etc.), several studies tested PAPP-A as a possible predictor for stillbirth, among other markers [18].

In 2002, Smith et al. conducted a study where the results of 8,839 pregnant women were analyzed [19]. The authors reported that women with first and early second trimester PAPP-A levels in the lowest 5th centile (adjusted odds ratio 3.6; 95% CI 1.2 - 11.0) were more likely to experience intrauterine death, pointing out that the IGF system has a key role in the pregnancy outcome. The same results were reported by Dugoff et al. in 2004 [20]. Out of 34,271 pregnancies between 10 weeks, three days and 13 weeks, six days of gestation, 95 women (0.28%) suffered intrauterine death before the 24th week (0.02 P-value). The authors concluded (among others) that first trimester low PAPP-A levels are associated with intrauterine fetal death at ≤ 24 weeks of gestation. The same results were obtained by Marleen et al. in 2014, reporting significantly lower PAPP-A levels in pregnancies resulting in intrauterine fetal death (0.25 MoM, interquartile range (IQR) 0.20 - 0.31; p 0.009) and by Kaijomaa et al. in 2017, where nine (0.9%) stillbirths were recorded among patients with low PAPP-A levels while none were recorded in the control group [21-22].

SGA - intrauterine growth restriction (IUGR)

An important parameter that made the correlation of PAPP-A to fetal development is (the proven to be accurate) pregnancy dating that first-trimester screening ensures. However, when strictly defining growth restriction under SGA less than the 5th centile, studies usually result in a proportion of neonates with a higher risk of adverse outcomes, in contrast to studies where SGA is set below the 10th centile. Accordingly, especially when used as a single marker, PAPP-A levels < 1st centile have a strong positive predictive value (PPV) for intrauterine growth restriction (IUGR), (15.8 % and 24% - 26% for SGA below the 5th and 10th centile, respectively) [16-17, 23].

On the contrary, reports of PAPP-A < 5th centile led many researchers to combine other indicators, even the second trimesters’ markers, to increase specificity and sensitivity. Such indicators usually consist of maternal risk factors, fetal routine parameters, Doppler measurements, and monitoring the development of first trimester maternal serum markers after the 14th week.

In 2006, Smith et al. presented a study of 8,483 women in order to associate maternal serum alpha-fetoprotein (AFP) and PAPP-A to adverse outcomes [24]. Combined, the AFP > 95th centile and PAPP-A < 5th centile levels have a 32.1 % possibility for SGA, suggesting mandatory monitoring for fetal development on women with such results.

Similarly, in order to evaluate if low first‐trimester serum PAPP‐A can accurately predict fetal growth, Fox et al. reported a study of 239 pregnant women out of which 25 had a low PAPP-A (< 5th centile), had undergone second trimester ultrasound examination, and who had at least one second trimester marker for fetal growth restriction (estimated fetal weight < 25th centile, a greater than seven-day growth delay compared to the established gestational age, or a ratio of head to abdominal circumference greater than 90th centile). The authors concluded that the rate of birth weight < 10th centile was 47.8% and the rate of birth weight < 5th centile was 21.7%, supporting that a poor perinatal outcome is quite possible for pregnancies with PAPP-A < 5th centile and with one second trimester fetal growth restriction [25].

Likewise, Cooper et al. reported in the same year a prospective interventional study in order to evaluate a possible correlation of adverse in pregnancies with low maternal serum PAPP-A level (< 0.4 MoM) and positive uterine artery Doppler (PI > 1.45) at 18 to 22 weeks of gestation [26]. Among these patients, the possibility of SGA was 36% at 18 weeks and 64% at 22 weeks [26].

Preeclampsia

Preeclampsia is a gestational disease that usually occurs during the third trimester of pregnancy. It is diagnosed clinically by maternal hypertension (> 140/90 mmHg) and proteinuria (> 300 mg/day), both mediated by angiogenic factors released into the maternal circulation, causing vasoconstriction due to placental dysfunction. Clinical manifestations of preeclampsia can occur during pregnancy or even after childbirth and can lead to fatal maternal and fetal complications without treatment [27].

Since PAPP-A is routinely used in first trimester screening and due to the need for an as early as possible detection of preeclampsia, most studies are conducted in the first trimester. However, some studies focused on preeclampsia prognosis using second and third trimester PAPP-A results.

First Trimester

Even though some reports concluded that PAPP-A levels of women developing preeclampsia showed no difference than the controls, many studies reported the opposite [28-30]. In 2002, the first multicenter study testing the relevance of first trimester PAPP-A to preeclampsia was published. Out of 8,839 women at eight to 14 weeks of gestation, the authors reported an increased risk of preeclampsia in the group with PAPP-A in the lowest 5th centile (adjusted odds ratio, 2.3; 95% CI 1.6 - 3.3) [19]. Similarly, in 2004, Dugoff et al. supported that women with PAPP-A under the 5th centile are more likely to experience preeclampsia among other adverse outcomes [28]. The same result was observed by Zwahlen et al. in 52 cases of preeclampsia and twice reported in 2009 by Poon et al. in 157 cases with decreased PAPP-A (0.53 MoM and 0.93 MoM) and in another 156 women who developed preeclampsia with low first trimester PAPP-A levels as well (0.555 MoM and 0.911 MoM) [20, 31-33]. Many other studies have also tested the reliability of PAPP-A as a predictor for preeclampsia with positive results. Either tested as a single predictor or combined to other markers, such as placental protein-13, placental growth factor, uterine artery pulsatility index, inhibin A, and others, PAPP-A proved to be a promising predictor [34-37]. 

Second Trimester

Not many studies about second trimester PAPP-A levels and preeclampsia are available in the literature. First, in 2003, Bersinger et al. reported decreased levels of maternal serum PAPP-A levels in the early second trimester in 53 women (with or without fetal growth restriction) compared to 65 controls but found no difference in the late second trimester. Similarly, in 2011, D’Anna et al. supported that the best period for preeclampsia prognosis might be at the beginning of the second trimester, describing a PAPP-A level in women with preeclampsia of < 0.3 MoM. On the contrary, in 2012, Bestwick et al. concluded that the correlation of early second trimester PAPP-A and preeclampsia could be derived comparing 77 women who developed preeclampsia to 224 controls [38-40].

Third Trimester

Since 1977, increased third trimester serum PAPP-A was associated with preeclampsia. However, there are no data currently supporting that PAPP-A could be a prognostic factor for preeclampsia because some studies conducted since then support that the difference in the serum PAPP-A is observed only in women with albuminuria (severe preeclampsia); others report no differences between serum PAPP-A levels of women suffering preeclampsia and controls, while others reveal increased concentration in the third trimester [41-46].

Preterm Birth

Pregnancy disorders, such as preeclampsia and intrauterine growth restriction, are frequent reasons for preterm delivery. Therefore, most of the studies up to date have tested the reliability of PAPP-A on the prognosis of the underlying disorder rather than predicting preterm delivery. To date, there are no studies proving an increased sensitivity or specificity of a single marker for early detection of preterm birth, and even though PAPP-A < 5th centile was reported in some cases to be possibly associated with preterm birth, no studies have currently proven a significant association of this marker with increased risk of preterm delivery (especially when using the marker MoMs as continuous variables) [16, 23].

Down Syndrome

Trisomy 21 is a maternal age-related genetic disorder that without prenatal diagnosis is estimated to be one in 800 spontaneous pregnancies. Owing to an increase in the mean age of pregnant women (one in 740 pregnancies in 1974 and one in 504 pregnancies with trisomy 21 in 1997), modern medicine led many high income-countries to provide first trimester pregnancy screening for Down syndrome [47].

In accordance with the International Prenatal Screening Research Group (IPSRG), Wald et al. presented PAPP-A as one of the two maternal serum markers with the greatest promise for trisomy 21 prediction [48]. With a median value of 0.38 MoM, PAPP-A is 60% lower in the maternal serum of pregnancies affected by Down syndrome.

With many scientists confirming this data, nowadays, screening between the 11th to 13th week of gestation has been established as one of the most financially appropriate and reliable prenatal screenings. PAPP-A combined with maternal age, nuchal translucency, and beta human chorionic gonadotropin (HCG) provides high detection rates for Down syndrome of up to 60% - 85% for combined first trimester prenatal screening (and up to 94% with the addition of inhibin A) with only a 5% false-positive rate [1, 49-50].

Combination of maternal serum markers

In order to estimate the diagnostic value, PAPP-A’s sensitivity and specificity were, among others, at the center of scientific attention being tested since the late 1970s. PAPP-A used as a single marker has proved to be of limited detection rate [33], but when combined to other markers, the sensitivity rate increases. In 2009, Akolekar et al. combined PAPP-A with inhibin A, activin A, mean arterial pressure, PP-13, soluble endoglin (sEng), pentraxin, Doppler pulsatility index (PI), placental growth factor (PIGF), and P selection, in a 33,602 patients study, resulting in 61%, 79%, and 91% detection rates for late, intermediate, and early onset preeclampsia, respectively [29], while in 2010, Wortelboer et al. tested PAPP-A with PIGF, a disintegrin and metalloprotease 12 (ADAM 12), and disintegrin and beta hCG and reported only a 44% preeclampsia prediction rate with a 5% false rate (Table 1) [34].

**Table 1:** Studies on PAPP-A and Adverse Pregnancy Outcomes aIUP: abnormal intrauterine pregnancy; CI: confidence interval; EP: ectopic pregnancy; FASTER: First- and Second-Trimester Evaluation of Risk trial; FGR: fetal growth restriction; hCG: human chorionic gonadotropin; IUGR: intrauterine growth restriction; MoM: multiple of the median: NPV: negative predictive value; NT:nuchal translucency; OR: odds ratio; PAPP-A: pregnancy-associated plasma protein A; PD: preterm delivery; PE: preeclampsia; PGF: placental growth factor; PL: pregnancy loss; PP-13: placental protein-13; PPV: positive predictive value; SA: spontaneous abortion; SB: stillbirth; SGA: small for gestational age; UA Doppler: uterine artery Doppler; UtA-PI: uterine artery pulsatility index; w: weeks

Adverse pregnancy outcome		Author	Year	Participants	PAPP-A Result				Ref. No
Ectopic pregnancy		Mueller et al.	2004	122 (43 EP)	Increased risk if < 0.02 MoM				[11]
		Beer et al.	2011	18 (9 EP)	20/40 significant peptides/total				[12]
		Rausch et al.	2011	200 (100 EP)	Limited diagnostic value as a single marker				[13]
		Daponte et al.	2005	50 (27 EP)	Unable to distinguish EP from aIUP				[14]
Pregnancy Loss		Goetzl et al.	2004	7,932 (75 PL)	< 0.29 MoM (5.4 OR)				[15]
		Dugoff et al.	2008	417 (315 < 24 w & 102 > 24 w)	Low PAPP-A associated with early loss (< 24 w)				[16]
		Scott et al.	2009	44,535	< 0.2 MoM increased risk of adverse outcome				[17]
Intrauterine Fetal Demise		Smith et al.	2002	8,839 (22 SB)	Adjusted OR 3.6; 95% CI, 1.2 – 11.0				[19]
		Dugoff et al.	2004	34,271	< 5^th^ centile increased risk for pregnancy loss				[20]
		Marleen et al.	2014	929 with PAPP-A < 0.4 MoM	Low levels in stillbirths. For every doubling of PAPP-A, stillbirth odds decreased by 53%. Low values for PE and FGR diagnosis				[21]
		Kaijomaa et al.	2017	961	Low PAPP-A associated with aneuploides, SA, PD, PE, SGA				[22]
SGA-IUGR		Scott et al.	2009	44,535 (32 SGA)	< 0.2 MoM increased risk of adverse outcome				[17]
		Krantz et al.	2004	8,012	< 5^th^ centile risk for IUGR				[23]
		Smith et al.	2006	8,483	Low PAPP-A, OR 1.9 for SGA				[24]
		Fox et al.	2009	239 (PAPP-A < 5^th^ centile)	10.5% third trimester SGA				[25]
		Cooper et al.	2009	5,359 (289 < 5^th^ centile)	PAPP-A combined to 22 w UA Doppler predicted SGA (OR = 8.24, p = 0.001)				[26]
Preeclampsia	1^st^ trimester	Smith et al.	2002	8,839	PAPP-A < 5^th^ centile at 8 - 14 w risk for PE (adjusted OR 2.3; 95% CI 1.6 - 3.3)				[19]
		Spencer et al.	2007	446 controls & 44 PE	PAPP-A combined with PP-13 does not predict early PE				[28]
		Di Lorenzo et al.	2012	2,118 (25 PE – 12/15:early/late onset)	MoM 1,00 (0,97 - 1.01) OR 1.02 P: 0.976. Early PE (OR 0.63 P: 0.658); late PE (OR 1.62 P: 0.640)				[30]
		Dugoff et al., (the FASTER Trial)	2004	34,271 (764 PE)		OR	95%	CI	[20]
					< 10^th^	1.34	1.07	1.67	
					< 5^th^	1.54	1.16	2.03	
					< 1^st^	1.79	1.04	3.10	
		Zwahlen et al.	2007	156 (52 PE)	Low PAPP-A combined with low placental lactogen, increased NT and increased inhibin A: increased risk for PE				[31]
		Poon et al. (A)	2009	7,797 (34 PE)	Early and late PE combined to decreased PAPP-A (0.53 MoM and 0.93 MoM)				[32]
		Poon et al. (B)	2009	156 (32 delivery < 34 w and 124 delivery > 34 w)	< 5^th^ centile in 21.9% of early and 6.5% of late PE and association between log UtA-PI MoM and log PAPP-A MoM (P = 0.001)				[33]
		Wortelboer et al.	2010	568 (88 PE)	Low PAPP-A (median 0.82 MoM, P < 0.02)				[34]
		Audibert et al.	2010	893 (40 PE)	PAPP-A combined with clinical characteristics, inhibin A and PGF = 75% detection of early-onset PE				[35]
		Kang et al.	2008	3076 (32 PE)	1^st^ trimester PAPP-A concentration was significantly lower and concentrations of early 2^nd^ trimester inhibin-A and hCG significantly elevated				[36]
		Nasrin et al.	2010	200 (3 PE)	PAPP-A sensitivity: 82%, specificity: 95%, PPV: 87%, NPV: 93%				[37]
	2^nd^ trimester	Bersinger et al.	2004	118 total - 53 PE (28 without FGR, 25 with FGR	PAPP-A reduced at 23 & 35 w of gestations with subsequent PE				[38]
		D’anna et al.	2011	602 (40 PE)	Possible detection of PE at 14 - 17 w				[39]
		Bestwick et al.	2012	301 (77 PE)	No use for early detection of 2nd trimester PE (0.97 MoM (95% confidence interval 0.73 to 1.25))				[40]
	3^rd^ trimester	Muravská et al.	2011	279 (35 PE)	Increased PAPP-A compared to controls (p < 0.05) - association of TT genotype of Cys327Cys polymorphism of the PAPP-A gene with preeclampsia				[41]
		Lin et al.	1977	88 (32 with toxemia - diastolic blood pressure greater than 110 )	Elevated PAPP-A in women with toxemia				[42]
		Hughes et al.	1980	272 (39 PE)	Elevated PAPP-A in PE patients				[43]
		Imaizumi et al.	1983	231 (44 pathological)	Elevated PAPP-A in PE patients				[44]
		Barnea et al.	1986	49 (13 hypertension – toxemia)	A higher placental concentration of PAPP-A in patients with hypertension than those with toxemia				[45]
		Atis et al.	2012	73 (36 PE)	PAPP-A level at last trimester increases in all mild-severe PE but not predictive for the severity of PE				[46]

## Conclusions

To date, the established association of low PAPP-A levels to Down syndrome has led to the wide use of PAPP-A measurement for the diagnosis and prognosis of prenatal adverse outcomes. Over the last decades, low maternal serum PAPP-A at 11 to 13 weeks of gestation has been tested with promising results as a prognostic factor for many adverse pregnancy outcomes. In particular, PAPP-A has shown great promise in the diagnosis and prognosis of Down syndrome, stillbirth, SGA, IUGR, and preeclampsia. Due to its particular features, ease of measurement and the variety of studies testing it, PAPP-A has become an integral part of prenatal control with data proving that this marker can be used either as a single predictor or combined with other maternal serum markers. So far, PAPP-A, beta HCG, maternal age, and nuchal translucency quantification are used as a mandatory protocol for prenatal screening in many high-income countries. In addition, the cut-off percentiles and multiple of the media are indicators of the pregnancy risks so that therapists are better able to provide a more careful morphological assessment, followed by stricter monitoring of fetal growth, and in order to inform and guide pregnant women on the possible pregnancy outcomes. PAPP-A has been an innovation in the last decade for prenatal screening; however, more studies are needed in order to achieve the best predicting values for specific structural abnormalities and genetic syndromes.
